# Case report: two human *Streptococcus suis* infections in Borneo, Sabah, Malaysia

**DOI:** 10.1186/s12879-017-2294-z

**Published:** 2017-03-04

**Authors:** Giri Shan Rajahram, Ahneez Abdul Hameed, Jayaram Menon, Timothy William, Paul Anantharajah Tambyah, Tsin Wen Yeo

**Affiliations:** 1 Infectious Diseases Unit, Department of Medicine, Queen Elizabeth Hospital, Kota Kinabalu, Sabah Malaysia; 2 Clinical Research Centre, Queen Elizabeth Hospital, Kota Kinabalu, Sabah Malaysia; 3 Microbiology Unit, Department of Pathology, Queen Elizabeth Hospital, Kota Kinabalu, Sabah Malaysia; 4 Department of Medicine, Queen Elizabeth Hospital, Kota Kinabalu, Sabah Malaysia; 5 Jesselton Medical Centre, Kota Kinabalu, Sabah Malaysia; 6 Department of Medicine, Yong Loo Lin School of Medicine, National University Health System, National University of Singapore, Singapore, Singapore; 7 Lee Kong Chian School of Medicine, Nanyang Technological University, Singapore, Singapore; 8 Institute of Infectious Disease and Epidemiology, Tan Tock Seng Hospital, Singapore, Singapore; 9 Global Health Division, Menzies School of Health Research, Darwin, Australia

**Keywords:** *Streptococcus suis*, Meningitis, Sabah, Case report

## Abstract

**Background:**

*Streptococcus Suis (S.suis) is* increasingly being recognised as a potentially preventable emerging zoonotic infection in humans with a global distribution. It is a major cause of meningitis especially among those in contact with pigs and has also been associated with a toxic shock syndrome.

**Case presentations:**

We report the first two human cases from Sabah, Borneo, Malaysia which expands the global reach of this important pathogen. Here, we illustrate their epidemiological risk factors, clinical presentation and resulting sequelae of both patients.

**Conclusion:**

The continued public health threat of zoonotic infections such as *S.suis*, highlights the need for accurate epidemiological surveillance, regulation of pig farming, slaughtering and continued advocacy of best practices for pork preparation and consumption.

## Background

*Streptococcus suis (S.suis)* is a porcine zoonotic infection which is increasingly recognised. Meningitis and septicaemia have been described as the most common clinical syndromes [1]. Since the first reported human case in Denmark, increasing numbers have been reported worldwide [2]. In South East Asia, Thailand and Vietnam have reported the largest number of cases with sporadic reports from Singapore, Philippines and Laos [2]. To date, there have not been any cases reported from Malaysia or Indonesia partly because of the limited pig rearing due to cultural reasons. We report two cases from Sabah, Borneo, Malaysia.

## Case report

The first patient was a 41-year-old previously healthy man who presented with 2 day history of fever, headache, vomiting, neck pain and behavioural change. He reared four pigs at home and had daily contact with them. On examination, he was confused and disorientated, with a Glasgow Coma Scale (GCS) 7/15, hypotensive with a blood pressure of 89/57 mmHg, and had meningeal signs, however there was no rash noted. He was intubated for airway protection, started on noradrenaline, ceftriaxone and acyclovir. Blood tests indicated leucocytosis (13.7 × 10^9^ white blood cell/L), neutrophilia (12.63 × 10^9^ neutrophils/L), lymphopenia (0.72 × 10^9^ lymphocytes/L), elevated C-reactive protein (320 mg/L), and hypoalbuminemia (28 g/L). Contrast enhanced computed tomography of the brain and transthoracic echocardiogram were normal. Lumbar puncture done 12 h after admission and initial antibiotic administration showed an opening pressure of 27 cmH_2_0 and the cerebrospinal fluid (CSF) was noted to be cloudy with a protein level of 2.16 g/L, a glucose level of 1.98 mmol/L (concomitant serum glucose was 7.6 mmol/L), and 95 leukocytes/μL (30% neutrophils and 65% lymphocytes). CSF gram stain and culture did not reveal any organism however blood cultures after 3 h of incubation were positive for gram positive cocci in chains. Culture specimens were plated on blood agar and after 18 h of incubation in 37 °C under aerobic conditions, the growth showed alpha haemolytic colonies which were catalase negative. These colonies were identified using matrix-assisted laser desorption ionization time-of-flight mass spectrometry (MALDI-TOF MS) VITEK® MS MALDI-TOF MS (bioMériux) as *Streptococcus suis* with a 99.9% probability score. Antibiotic susceptibility testing was carried out according to the Clinical Science Laboratory Institute [3] testing recommendation using the Kirby-Bauer method. The strain was sensitive to penicillin and ceftriaxone the minimal inhibitory concentration (MIC) of penicillin and ceftriaxone were identified by epsilometer test (E-test bioMériux) with an MIC <0.12 ug/ml and <1.0ug/ml respectively). The patient was extubated after 48 h and continued to make a good clinical recovery with antibiotics and completed a two week course of ceftriaxone. However, in the second week of admission he developed tinnitus with reduced hearing in both ear associated with vertigo, loss of balance and nausea. He was scheduled for a pure tone audiometry on clinic follow up upon discharge however defaulted his appointment and we do not have any record of permanent eighth nerve damage.

The second patient was a 44-year-old man, presented with a 2 day history of fever with chills and rigors associated with headache and vomiting. On examination he was alert and conscious with no signs of meningism. He had no fever on admission, the blood pressure was 124/68 mmHg and heart rate 81 beats per minute. Systemic examination was unremarkable. He worked as a butcher in local market handling pork and had injured his thumb while slaughtering a pig two days prior to onset of symptoms. Blood tests showed leucocytosis (35.4 × 10^9^ white blood cell/L), neutrophilia (33.04 × 10^9^ neutrophils/L), lymphopenia (0.70 × 10^9^ lymphocytes/L), elevated C-reactive protein (226.8 mg/L), and hypoalbuminemia (29 g/L). Blood culture on admission and antibiotic susceptibility testing performed by methods described above was positive for *Streptococcus suis* which was sensitive to penicillin. Lumbar puncture performed 48 h after admission was normal with no white blood cells and normal glucose and protein. His echocardiogram also did not show any evidence of endocarditis. He completed two weeks of intravenous penicillin G and made complete recovery on discharge without any residual neurological or otological deficits.

## Discussion

Consistent with previous reports [1, 2, 4] both our patients had significant exposure to pigs prior to developing infection. In Asia, besides direct trauma as with our second patient, it is increasing recognised that suboptimal preparation and consumption of pork and its organs may increase the risk of acquiring this disease [1, 2, 4].

The first patient presented with meningitis associated with bacteraemia and developed hearing loss and vestibular symptoms during the course of admission. This is consistent with previous reports of *S. suis* meningitis with labyrinthitis [5] and sensory neural hearing loss occurring in up to 2/3rd of patients with a guarded prognosis for recovery [4]. The use of dexamethasone therapy in association to reducing hearing loss is controversial, with one study showing reduction in severe hearing loss [4] and a subsequent case series demonstrating hearing loss continued to occur despite therapy [6]. As with other forms of bacterial meningitis, the best outcomes are likely to be obtained by early recognition and rapid administration of parenteral antibiotics, combined administration of dexamethasone patients with a history of porcine exposure, and in regions with a high prevalence of *S.suis* meningitis [7]. Referral to the Otorhinolaryngologist for audiometry and vestibular testing may be needed including early evaluation for cochlear implant [8].

Although meningitis is the most common described presentation of *S.suis* infection, sepsis, uveitis, endoptahlmitis, endocarditis, arthritis and spondylodiscitis have all been previously reported [1, 2]. Of note, large outbreaks in China have described streptococcal toxic shock syndrome (STSS) associated with *S. suis* infection with rapid clinical deterioration including purpura fulminans and high mortality [9, 10]. While this syndrome has not been recognised outside of China, there is a concern that limited diagnostic resources and a lack of accessibility to healthcare may be the reason why this is so in other parts of Asia.

To date, 29 serotypes *S.suis* have been described [11]. The majority of human cases have been attributed to serotype 2 with also some sporadic cases from other serotypes, mainly serotype 14^10^. Reliable multiplex PCR methods are replacing traditional biochemical based methods in detecting these serotype [12], this coupled with multilocus sequence typing (MLST) this has led to better epidemiological characterization and improved understanding virulence patterns of this organism [11]. Unfortunately, these were not available in our regional hospital as the need for specialised expertise and funding limits such testing to regional and national reference laboratories or in an academic setting.

## Conclusion

In summary our cases illustrate the common clinical presentation and sequelae associated with *Streptococcus suis* infection. Although it is a common zoonotic infection in certain parts of Asia, the true burden of illness in Malaysia in not know. The continued public health threat of zoonotic infections such as *S.suis*, highlights the need for accurate epidemiological surveillance, regulation of pig farming, slaughtering and continued advocacy of best practices for pork preparation and consumption.

## Abbreviations

CSF: Cerebrospinal fluid
MALDI-TOF MS: Matrix-assisted laser desorption ionization time-of-flight mass spectrometry
MIC: Minimal inhibitory concentration
MLST: Multilocus sequence typing
*S.suis*: *Streptococcus suis*
STSS: Streptococcal toxic shock syndrome
